# Implementing Infection Control and Quality of Life Best Practices in Nursing Homes With Project ECHO: Protocol for a Patient-Centered Randomized Controlled Trial

**DOI:** 10.2196/34480

**Published:** 2022-05-13

**Authors:** William A Calo, Erica Francis, Lan Kong, Ruth Hogentogler, Emily Heilbrunn, Abbey Fisher, Nancy Hood, Jennifer Kraschnewski

**Affiliations:** 1 Department of Public Health Sciences Penn State College of Medicine Penn State University Hershey, PA United States; 2 Department of Medicine College of Medicine Penn State University Hershey, PA United States; 3 Health Sciences Center University of New Mexico Albuquerque, NM United States

**Keywords:** infection control, COVID-19, nursing home, telementoring, Project ECHO, case-based learning, patient-centered outcome, RE-AIM, randomized controlled trial, implementation, quality of life, best practice, guideline, comparison, effectiveness, intervention

## Abstract

**Background:**

Nursing homes in the United States were devastated by COVID-19, with 710,000 cases and 138,000 deaths nationally through October 2021. Although facilities are required to have infection control staff, only 3% of designated infection preventionists have taken a basic infection control course prior to the COVID-19 pandemic. Most research has focused on infection control in the acute care setting. However, little is known about the implementation of infection control practices and effective interventions in nursing homes. This study utilizes Project ECHO (Extension for Community Health Outcomes), an evidence-based telementoring model, to connect Penn State University subject matter experts with nursing home staff and administrators to proactively support evidence-based infection control guideline implementation.

**Objective:**

Our study seeks to answer the research question of how evidence-based infection control guidelines can be implemented effectively in nursing homes, including comparing the effectiveness of two ECHO-delivered training interventions on key patient-centered outcomes such as reducing the number of residents with a COVID-19 diagnosis.

**Methods:**

A stratified cluster randomized design was utilized. Using a 1:1 ratio, we randomly assigned 136 nursing homes to ECHO or ECHO Plus arms. Randomization was stratified by geographic location, baseline COVID-19 infection rate, and facility capacity. The study had two phases. In phase one, completed in July 2021, nursing homes in both study arms received a 16-week infectious disease and quality improvement training intervention via real-time, interactive videoconferencing and the ECHO learning model. Phase one sessions were up to 90 minutes in duration. In phase two, completed in November 2021, the ECHO group was offered optional 60-minute office hours for 9 weeks and the ECHO Plus group received 9 weeks of 60-minute sessions on emerging topics and an additional 8-session refresher series on infection control.

**Results:**

A total of 290 nursing home facilities were assessed for eligibility, with 136 nursing homes recruited and randomly assigned to ECHO or ECHO Plus. Guided by the Reach, Effectiveness, Adoption, Implementation, and Maintenance (RE-AIM) framework, we will simultaneously evaluate the study’s effectiveness and implementation outcomes at baseline (intervention start date), and at 4, 6, 12, and 18 months. The primary outcome is the COVID-19 infection rate in nursing homes. Secondary outcomes include COVID-19 hospitalizations and deaths, flu-like illness, and quality of life. Surveys and interviews with participants will also provide data as to the adoption, implementation, and maintenance of best practices taught throughout ECHO sessions.

**Conclusions:**

A multipronged approach to improving infection control and emergency preparedness in nursing homes is important, given the toll that the COVID-19 pandemic has taken on residents and staff. The ECHO model has significant strengths when compared to traditional training, as it allows for remote learning delivered by a multidisciplinary team of experts, and utilizes case discussions that match the context and capacity of nursing homes.

**Trial Registration:**

ClinicalTrials.gov NCT04499391; https://clinicaltrials.gov/ct2/show/NCT04499391

## Introduction

Nursing homes were ground zero for the COVID-19 pandemic; over half of US nursing home residents were infected (710,000) and 1 in 10 (138,000) died of COVID-19 between March 2020 and October 2021 [1]. The COVID-19 pandemic shines a spotlight on the infection control principles for some of the frailest, most vulnerable individuals in the United States [2]. However, infectious disease outbreaks in nursing homes are not a new challenge, including organisms such as norovirus, influenza virus, and *Streptococcus* [3]. Fortunately, 85.8% of US nursing home residents have been vaccinated against COVID-19, although vaccination alone is not sufficient to prevent outbreaks in the nursing home setting [1].

There are several reasons that infection control within the nursing home setting is a major challenge. Although facilities are required to have designated infection control staff, only 3% had taken a basic infection control course before the COVID pandemic [4-6]. A cross-sectional survey of randomly selected US nursing homes assessed their adherence to the Centers for Medicare and Medicaid Services’ (CMS) final rule requiring these facilities to develop an infection control program, employing a trained infection preventionist. One-third of the sample (n=990) reported receiving an infection control deficiency citation [7]. Other challenges to infection control include caregivers moving frequently between rooms, inconsistent hand washing, and vaccine hesitancy [7]. This highlights the need for a multipronged approach to infection control in nursing homes, which is key to battling COVID-19 and future infectious outbreaks in nursing homes.

The Centers for Disease Control and Prevention (CDC) outlines several strategies to assist nursing homes in addressing the pandemic [8]. Unfortunately, these evidence-based infection control practices have failed to translate into effective implementation [9]. Although guidelines may appear relatively straightforward, implementation requires organizational capacity, staff engagement, and problem-solving that can strain organizations lacking appropriate training, resources, and support. Identifying effective implementation for evidence-based practices is of critical importance to decision makers in addressing infection control in nursing homes and requires studying innovative approaches. Although significant research has focused on infection control in the acute care setting, little is known about the implementation of practices and effective interventions in nursing homes [7,10]. A recent systematic review [11] on the effectiveness of preventing or reducing COVID-19 in nursing homes found little evidence linking interventions or strategies to robust data on effectiveness. Most of these studies were observational, with no randomized controlled trials (RCTs) reported to date.

This protocol describes a stratified cluster randomized design to evaluate an intervention utilizing Project ECHO (Extension for Community Health Outcomes), an evidence-based telementoring model to connect Penn State University infectious disease, geriatric, and nursing home experts with remote nursing home staff and administrators to proactively support evidence-based infection control guideline implementation. Our study seeks to answer the research question of how evidence-based infection control guidelines can be implemented effectively in nursing homes. Our primary aim was to compare the effectiveness of a 16-week infectious disease and quality improvement curriculum [12] (ECHO) versus ECHO *plus* a 9-week series on emerging COVID-19 topics and an 8-week infection control refresher series (ECHO Plus) on the number of nursing home residents with COVID-19. Our secondary aims were to compare the effectiveness of ECHO versus ECHO Plus on other patient-centered outcomes (COVID-19 hospitalizations and deaths, flu-like illness, and quality of life [QOL]) and evaluate the impact of intervention conditions on key implementation outcomes in nursing home facilities based on the Reach, Effectiveness, Adoption, Implementation, and Maintenance (RE-AIM) framework.

## Methods

### Ethics Approval and Informed Consent

Approval for this study has been obtained from the Penn State Institutional Review Board at the Penn State College of Medicine in Hershey, Pennsylvania (STUDY00015883). All participants received information about the study and were asked to give consent before participating in the study.

### Study Design

A stratified cluster randomized design was used. According to a 1:1 ratio, we randomly assigned 136 nursing homes (with approximately 16,700 residents) to ECHO or ECHO Plus. Randomization was stratified by geographic location (rural vs urban), baseline COVID-19 infection rate (some vs none), and facility capacity (<60 beds vs ≥60 beds). Patient-centered outcomes, including nursing home residents with COVID-19 infections, flu-like illness, COVID-19 hospitalizations, deaths, and QOL, were assessed at baseline (intervention start date), and at 4, 6, 12, and 18 months. Our study is guided by the RE-AIM framework to critically evaluate both effectiveness and implementation outcomes of the proposed cluster RCT [13].

### Recruitment

National nursing home lists were obtained using CMS data, state agency and nursing home association contact websites, and engaged stakeholders. We assessed 290 nursing homes for eligibility from national nursing home lists comprising a total of 2000 facilities, focusing primarily in Pennsylvania, but including other states in the Northeast and Midwest, including Connecticut, Delaware, Illinois, Indiana, Maryland, New Hampshire, New Jersey, New York, Ohio, Vermont, Virginia, and Wisconsin. Recruitment occurred from December 2020 to February 2021. In Pennsylvania, nursing homes were recruited through collaborations with key stakeholders, including state agencies and state nursing home organizations, through phone calls and emails. For nursing homes located in other states, we utilized lists obtained from Project ECHO at the University of New Mexico, and made phone calls and sent emails to either the director of nursing or nursing home administrator. Inclusion criteria included being a CMS-eligible facility and no prior participation in a prior ECHO-delivered COVID-19 intervention. Eligible nursing homes were asked to identify two nursing home staff members to participate in the study, and we encouraged participation by infection control staff and other facility leadership (eg, medical director, director of nursing, administrators).

### Intervention

The intervention for this study included the Agency for Healthcare Research and Quality (AHRQ) ECHO National Nursing Home COVID-19 Action Network [12], supported by AHRQ and in collaboration with Project ECHO at the University of New Mexico Health Sciences Center and the Institute for Healthcare Improvement (IHI). This network provided training and mentorship to nursing homes across the country to increase the implementation of evidence-based infection prevention and safety practices to protect residents and staff. Using the Project ECHO model of telementoring, all nursing homes received the intervention in two sequential phases from December 2020 to November 2021 (Table 1).

During each session, nursing home staff participated in remote sessions led by a multidisciplinary specialist team consisting of subject matter experts from the following areas of expertise: emergency preparedness, nursing home operations, infectious disease and infection control, geriatrics, mental health, and quality improvement.

To incentivize participation, continuing education credits were awarded for attending live sessions. In addition, a stipend of US $6000 was available through the AHRQ initiative to eligible facilities that participated in or viewed recordings for 13 out of 16 sessions in phase one.

**Table 1:** Summary of comparators.

Study phase		ECHO^a^	ECHO Plus
**Phase one**			
	16-week infection control ECHO	✓	✓
	Quality Improvement component	✓	✓
**Phase two**			
	Nine-week office hours	✓ (optional)	
	Nine-week ECHO		✓
	Eight-week refresher series (fall 2021)		✓

^a^ECHO: Extension for Community Health Outcomes.

### Phase One

#### Overview

Nursing homes in both study arms received the 16-week AHRQ ECHO National Nursing Home COVID-19 Action Network (Network) curriculum via real-time, interactive videoconferencing using Zoom at no cost to participants. The curriculum was developed specifically for this intervention in partnership between AHRQ, the University of New Mexico’s ECHO Institute, and the IHI [12]. Session recordings were available for those who were unable to participate live. Phase one sessions were up to 90 minutes in duration and held weekly for 4 months (16 sessions total) at regularly scheduled times. All sessions followed the required program format for the Network as detailed below.

#### Introductions

Introductions (5 minutes) provide an inviting atmosphere, with participants including nursing home staff at the frontline in caring for patients and overseeing infection control policies and operations.

#### Didactic Presentations

Subject matter experts deliver two presentations of 10-15 minutes each on the week’s topic (Table 2), including a quality improvement topic delivered by an IHI expert.

**Table 2:** Phase one curriculum topics.

Week	Topic
1	Preventing and limiting the spread of COVID-19 in nursing homes
2	Infection prevention and management: guidance and practical approaches for use of personal protective equipment during COVID-19
3	COVID-19 vaccine information and rollout
4	Vaccine wrap-up and infection prevention and management: promoting solutions for making the built environment safer, and guidance for cleaning and disinfecting
5	The role of certified nursing assistants in managing and supporting residents and families during COVID-19
6	Managing social isolation during COVID-19: perspectives on staff and residents
7	Infection prevention and management: approaches to cohorting during COVID-19
8	Promoting safe care transitions during COVID-19 –admissions, discharges, and transfers
9	Supporting the emotional well-being of staff caring for residents during COVID-19
10	COVID-19 community transmission and nursing home screening strategies
11	Advance care planning in the time of COVID-19
12	COVID-19 testing for nursing homes
13	Promoting safe visitation and nursing home reopening during COVID-19
14	Staff returning to work safely during COVID-19
15	Interprofessional team management of mild cases of COVID-19
16	Effective leadership and communication during COVID-19

#### Case Presentations

Typically, each session includes case-based discussions (1-2 cases/session, 30 minutes) to ensure mastery of the content and skills. Each participant presents at least one case during the program. Other participants are encouraged to ask clarifying questions and weigh in on recommendations, followed by ECHO experts who provide advice on addressing each case using best practices. Recommendations are summarized verbally during the session and sent via email. To protect patient confidentiality, cases are presented without protected health information using a standard REDCap case form.

#### Question and Answer Period

Participants were invited to join an optional question-and-answer session (30 minutes) to further discuss curriculum topics or new challenges in nursing homes.

#### Close and Debrief

All sessions conclude (5 minutes) with a reminder to complete the session evaluation as provided by the AHRQ, and the subject matter experts encourage participants to put into practice what they have learned, which is later assessed.

### Phase Two

The ECHO Plus group received 9 weeks of live 60-minute ECHO sessions instead of office hours, following the format described for phase one and covering emerging topics (Table 3) developed by the research team specifically for this intervention. These topics were identified as timely and important by our stakeholders, subject matter experts, and feedback from participating nursing homes. If nursing home staff were unable to attend the session live, they were offered the recording of that session. In addition, ECHO Plus facilities received an additional 8-session refresher series running from September to November 2021, providing an opportunity to further cover topics that are part of the CDC infection control training and prioritized by our stakeholders and nursing home participants.

The ECHO group was offered the option to participate in phase two of the AHRQ ECHO National Nursing Home COVID-19 Action Network, which consisted of nine weekly 60-minute office hours, in which participants could drop in on an as-needed basis to ask specific questions and receive guidance from our experts on a variety of topics. Although the ECHO group did not receive a brief lecture, we ensured that all session resources shared with the ECHO Plus group, including PowerPoint presentations, were made available to them in a shared online folder.

**Table 3:** Phase two curriculum topics for ECHO Plus.

Week	Nine-week emerging topic series	Eight-week refresher series
1	COVID-19 variants and vaccine hesitancy	Monoclonal treatment, updates on variants/visitation, flu season and COVID-19
2	Crisis management and communication	Booster updates/new guidelines, vaccines versus natural infection/long COVID, vaccine mandates for staff (impact on staffing), vaccine myths
3	Resident quality of life/social isolation	Navigating and interpreting regulatory and nonregulatory state DOH^a^ and federal CMS^b^/CDC^c^ guidelines: What is a “must” versus a “should”
4	Grief and loss (for staff, residents, and resident families)	Rounding, audits/checklists, staff onboarding, and training
5	Role of the medical director	Social isolation and grief refresher: trauma-informed care for residents and staff
6	Other staff roles (activities, facilities management, dining/food services)	Occupational Safety and Health Administration compliance training (including volunteers and contractors)
7	COVID-19 updates (information on boosters, new data, new guidance)	Emergency preparedness: now and in the future, nursing facilities as part of US critical infrastructure
8	Sustainability of best practices	Staff and leadership burnout and turnover; institutional knowledge
9	What’s next? Ongoing quality Improvement	N/A^d^

^a^DOH: Department of Health.

^b^CMS: Centers for Medicare and Medicaid Services.

^c^CDC: Centers for Disease Control and Prevention.

^d^N/A: not applicable.

### Stakeholder Engagement

**Figure 1:**
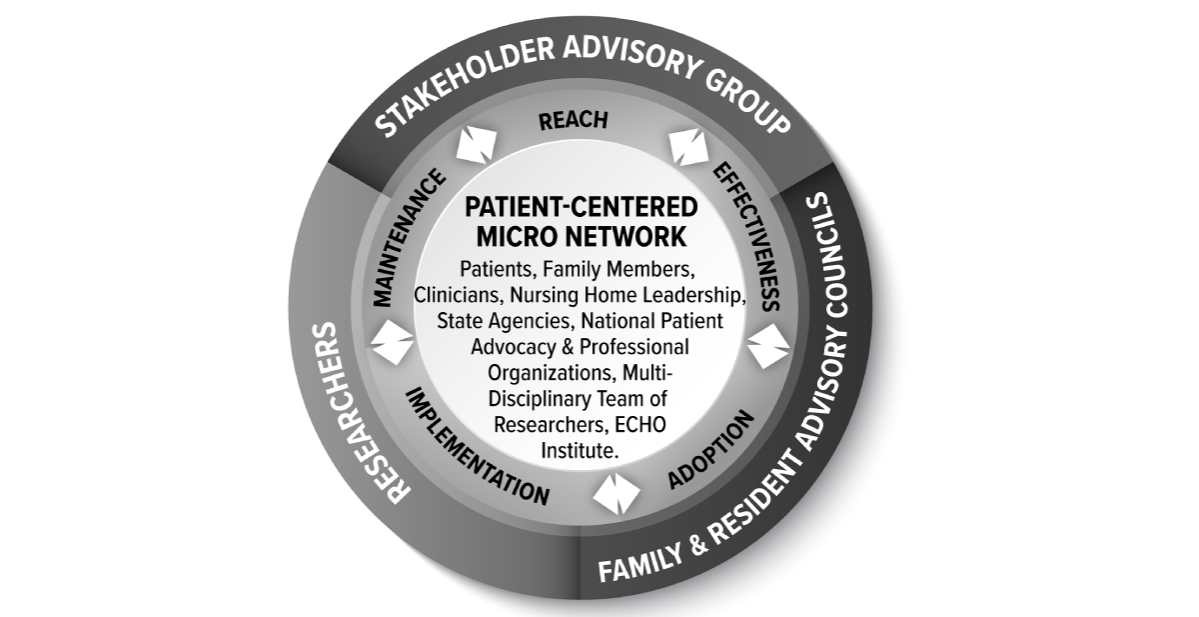
Patient-centered micro network.

We strengthened the patient-centeredness of the study with the addition of a stakeholder advisory board (Figure 1), including engagement on multiple levels in the proposed study’s planning and design. The stakeholder advisory board meets every other month, and once annually for a longer meeting, including nursing home patients and their families, nursing home staff/administration, state and federal policymakers, payers, and state professional organizations. These synergistic partnerships across all facets of nursing home care will ensure that our research continually focuses on what matters to stakeholders. We have engaged each of our stakeholders in development of this protocol and their input has shaped the study design. Stakeholders weigh in on important aspects of the study, including recruitment, data collection and analysis, and dissemination.

### Proposed Outcomes

Guided by the RE-AIM framework [13], we will simultaneously evaluate the study effectiveness and implementation outcomes (Table 4). We will evaluate different aspects of implementation practices in nursing homes. These outcomes will be assessed at baseline (intervention start date), and at 4, 6, 12, and 18 months. These data will be collected using publicly available data sets maintained by federal and state health agencies, validated tools adapted to this project, and interviews with nursing home staff.

The primary outcome is the COVID-19 infection rate in nursing homes (effectiveness). Deidentified patient data will be obtained using the Nursing Home COVID-19 Public File [1] along with three secondary outcomes: flu-like illness, COVID-19 hospitalizations, and deaths. Specifically, the variables that will be assessed include resident weekly and total admissions, resident weekly and total COVID-19 deaths, number of residents with new influenza, and number of residents with acute respiratory illness symptoms excluding COVID-19 and/or influenza. These data are also available for staff. Nursing home resident QOL will be measured using CMS’ Minimum Data Set [14], including emotional, symptom and functional statuses, behavioral disturbances, social support, patient engagement, and shared decision-making. CMS data will be linked to participating nursing homes.

Using the RE-AIM framework [13], we will collect several measures to assess how ECHO and ECHO Plus are utilized in nursing homes as implementation outcomes. We will measure reach by assessing the characteristics of nursing homes and staff who participate in the study and those who do not. We will then compare these data to the overall characteristics of nursing homes to assess representativeness. To accomplish this, we will record and categorize participation in each session (eg, number of staff per nursing home site, role of participants in nursing homes, engagement in sessions). At the beginning of each ECHO session, we ask that all participants place their name, email address, and affiliation in the chat box. In addition, we pull Zoom reports to identify participants and the length of time they joined each session (to ensure full participation). We also offer continuing education credits, which is a second opportunity to record engagement and assess quality dimensions that explain participation (eg, satisfaction, acceptability). We evaluate adoption by assessing the characteristics of adopters, as well as barriers and facilitators, using a set of validated instruments adapted to this project, including measures to assess inner and outer contexts of nursing homes [15], the Organizational Readiness for Implementing Change (ORIC) scale [16], the Practice Adaptive Reserve (PAR) scale [17], and the Change Process Capacity Questionnaire (CPCQ) [18].

**Table 4:** Proposed study outcomes mapped to the Reach, Effectiveness, Adoption, Implementation, and Maintenance (RE-AIM) framework.

Study outcomes	Description	Data source (timing of assessment)
Reach	Absolute number, proportion, and representativeness of nursing homes and staff who *agree to participate*	Study recruitment logs and staff survey (baseline)
Effectiveness	COVID-19 infection rate (*primary outcome*); flu-like illness, hospitalizations, deaths, quality of life (*secondary outcomes*)	National COVID-19 Nursing Home data file and CMS^a^ Minimum Data Set (baseline, 4, 6, 12, 18 months)
Adoption	Absolute number, proportion, and representativeness of nursing homes and staff who *initiate* and *complete* the ECHO^b^ series, and barriers and facilitators for adoption	Study participation logs; staff survey (baseline, 6 months) with validated measures, including ORIC^c^, PAR^d^, and CPCQ^e^; and key informant interviews (6 months)
Implementation	Nursing home staff knowledge and attitudes toward the various intervention functions and components, their level of implementation, and barriers and facilitators for implementation	Selected items from the CDC^f^ Preparedness Checklist; staff surveys (baseline, 6, 12 months) with validated measures; and key informant interviews (6 months)
Maintenance	Extent to which implemented guidelines for emergency preparedness in an infectious disease outbreak become part of nursing home policies postintervention	Key informant interviews (12 months)

^a^CMS: Centers for Medicare and Medicaid Services.

^b^ECHO: Extension for Community Health Outcomes.

^c^ORIC: Organizational Readiness for Implementing Change scale.

^d^PAR: Practice Adaptive Reserve scale.

^e^CPCQ: Change Process Capacity Questionnaire.

^f^CDC: Centers for Disease Control and Prevention.

Implementation will be assessed in nursing homes (enactment fidelity) using CDC’s COVID-19 Preparedness Checklist [19] for nursing homes as well as barriers and facilitators (ie, Implementation Climate questionnaire, Key Driver Implementation scale) [20]. For maintenance, we will assess policies nursing homes utilize to incorporate best-practice guidelines on addressing COVID-19, quality improvement, and infection control into routine practice. All staff survey data on implementation outcomes will be collected through REDCap. Our implementation evaluation will also include key informant interviews with a subsample of nursing home administrators and staff (n=30) following an explanatory-sequential design. With this design, our team will use interview discussions to further explain the effectiveness results of the study (infection rates, hospitalizations, and deaths) in the words of the implementers themselves, as well as strategies being implemented to support maintenance. These interviews will also help the study team understand the evolution of contextual factors that were not present at the beginning of the trial (eg, COVID-19 vaccine rollout in nursing home facilities). Main guiding questions (probing questions will be added as needed) are organized under three major themes (Textbox 1).

**Interview guide (6 months).**

**Theme 1: Experience with COVID-19**

Q1. How did the infrastructure of your organization (size, networks, or physical layout) affect the study outcomes?

Q2. Did you have sufficient resources to implement and administer the strategies presented in ECHO sessions?

Q3. Do you consider that the participation of your nursing home facility in the study was a success or a burden? Why?

**Theme 2: ECHO Intervention**

Q4. How does the intervention compare to other alternatives that may have been considered or that you know about?

Q5. What is your perception on the quality of the ECHO sessions and supporting materials? Did they meet your expectations?

Q6. Tell me a new strategy that your facility implemented in the past 6 months because of participating in this study.

**Theme 3: Vaccine Rollout**

Q7. Describe your facility’s experience with the COVID-19 vaccine rollout. What are you doing to ensure that new residents get vaccinated?

Q8. What is your facility doing to increase vaccine confidence and uptake among staff?

### Power Analysis

According to the CMS weekly data as of April 2021, the average weekly COVID-19 infection rate was 0.1%, the average number of residents in nursing homes (cluster size) was around 75-80, and the coefficient of variation of cluster size was approximately 0.76. Assuming a 1% infection rate in the ECHO arm over the 9 weeks when additional topic sessions are provided in the ECHO Plus arm, our study will have 80% power to detect a significant intervention effect if the infection rate is reduced to below 0.3% in the ECHO Plus arm. This calculation was based on a two-sided statistical test of difference between Poisson rates at α=.05 and an intracluster correlation coefficient≤0.01.

### Data Analysis

#### Overview

We will test the effectiveness of the ECHO Plus group by performing both individual-level analyses and cluster-level (nursing homes) analyses following the intention-to-treat principle. All statistical tests will be two-sided, with P<.05 considered statistically significant. We will compare the observed confounders between two study arms and adjust them in the analysis if they are not balanced by design. To account for intracluster correlations within nursing homes, we will use multilevel models such as mixed effects models or marginal models based on the generalized estimating equation (GEE) method to estimate the intervention effect. Outcomes at 6 months will be analyzed using generalized linear models, with an appropriate link function depending on outcome type. The intervention effect on infection risk will be estimated as the odds ratio or incidence rate ratio based on logistic, Poisson, or negative binomial regressions. State and cohort variations will be examined and accounted for using fixed or random effects in the models. Characteristics of residents (eg, age, gender, cognitive function/dementia) and nursing homes (eg, size, quality, baseline infection rate) will be adjusted. Using all measures at baseline, and at 4, 6, 12, and 18 months, we will perform longitudinal analysis to evaluate if the intervention effect changes over time using the same modeling approach but adding additional variables for time and time-by-intervention interaction. Correlations of repeated measures for the same resident will be considered in model estimation.

For cluster-level analyses, since the aggregated outcomes (infection, hospitalization) are rates or proportions between 0 and 1 instead of being binary or count variables, we will use β regression to model them with a logit link function so that the coefficient can be interpreted as a log odds ratio. We will also perform longitudinal analysis for aggregated outcomes based on β regression.

#### Subgroup Analysis

We hypothesize that the ECHO Plus effect is heterogeneous and expect stronger intervention effects in subgroups without cognitive dysfunction/dementia as well as in those who are younger. Subgroup analysis regarding key participant factors will be performed similarly to examine potential heterogeneity of intervention effects, further tested by interaction analysis. If the data are sufficient, we plan to explore the heterogeneity of the intervention effect on infection rate based on the key participant factors such as age group, gender, dementia, insurance status, and race/ethnicity.

#### Missing Data

We plan to record and report all reasons for dropout and missing data. Both mixed effects models and marginal models based on GEE methods are valid if the outcome data are missing completely at random. For data missing at random (ie, depends on observed data only), mixed models can still provide valid inference and the weighted GEE methods will be considered to account for this type of missing data. We will also conduct sensitivity analyses to examine the robustness of results to missing data. Multiple imputation methods will be used to address the missing data in the covariates.

## Results

**Figure 2:**
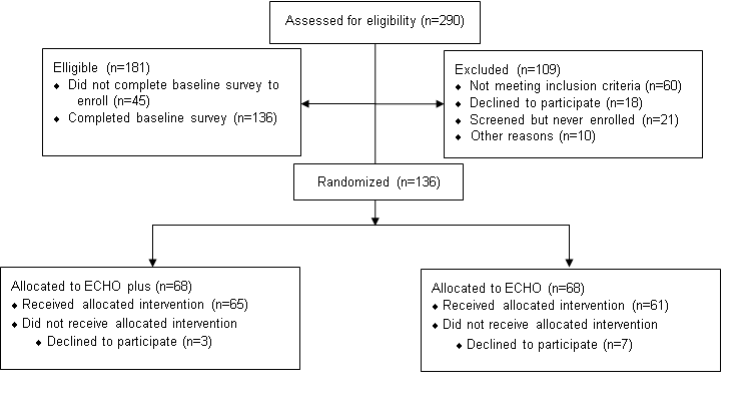
CONSORT (Consolidated Standards of Reporting Trials) flow diagram.

We assessed 290 nursing home facilities for eligibility and 136 nursing homes were enrolled and randomly assigned to ECHO or ECHO Plus (Figure 2). Phase one of the study was completed in July 2021 and phase two was completed in November 2021. Only six nursing home facilities dropped out.

**Figure 3:**
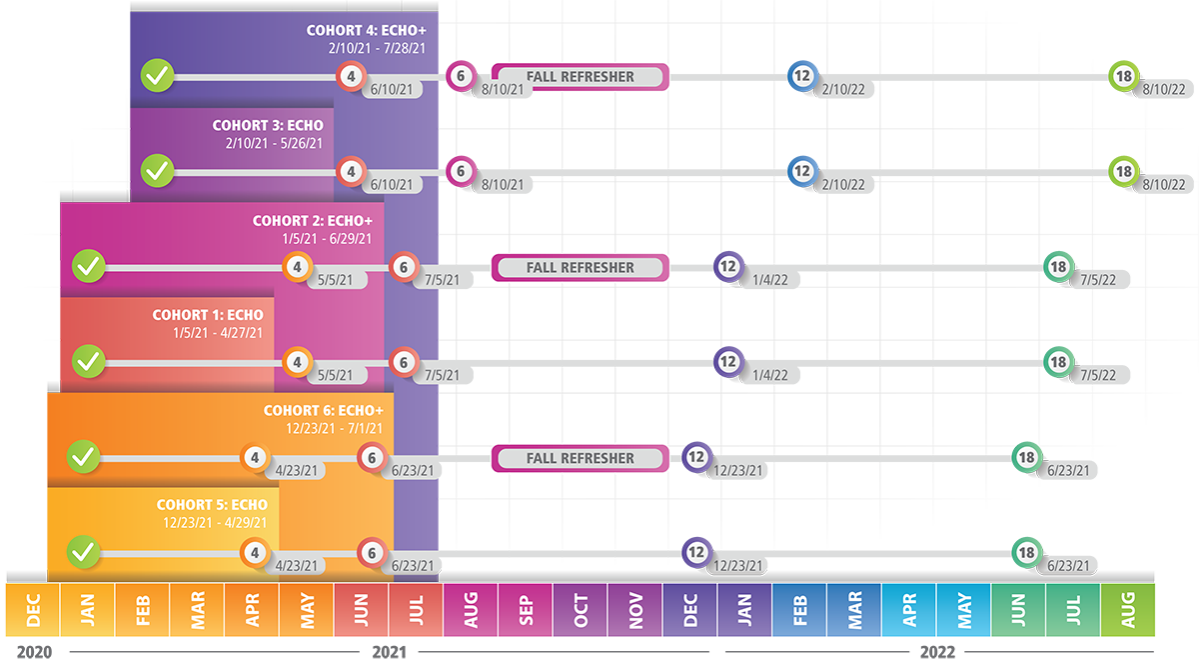
Study timeline.

Participating nursing home facilities were divided into six cohorts of up to 25 facilities each (Figure 3). This size allowed for maximum engagement in discussions during sessions. Further, as of April 2022, we have completed most interim assessments of primary and secondary outcomes, and we expect to finalize all data collection activities in August 2022, including our primary outcome evaluation at 18 months postintervention.

## Discussion

Our study was part of the larger AHRQ ECHO National Nursing Home COVID-19 Action Network. The 136 nursing homes enrolled in our study were among the 9058 sites in all 50 states, the District of Columbia, and Puerto Rico that benefited from no-cost training and mentorship to better protect their residents and staff against COVID-19. All of our intervention activities were completed in November 2021 and we expect to conclude our data collection activities in August 2022, which is 18 months postintervention. We hypothesize that nursing homes engaged in the ECHO Plus intervention will have fewer COVID-19 infections than those in the ECHO intervention after 18 months. Similarly, we hypothesize that nursing home participation in ECHO Plus will significantly improve QOL and decrease COVID-19 hospitalizations and deaths compared to the ECHO intervention.

Although little evidence was available on how to address COVID-19 in nursing homes, our study protocol was informed by formative work we conducted at the beginning of the pandemic. We conducted a needs assessment in April 2020 with nursing home administrators and staff (n=71) that indicated several challenges to implementing infection control strategies, including lack of infection control training, managing resident transfers, preventing transmissions, information overload, and staff well-being. We also launched a pilot COVID-19 ECHO series for health care providers, which engaged 16 nursing homes in Pennsylvania. This formative work demonstrated our team’s existing infrastructure and ability to rapidly recruit and engage nursing homes in research studies and provided a critical foundation to this protocol.

If we find our intervention to be effective, we strongly believe this work has great potential for dissemination and implementation. First, we are partnering with the leading institution of the ECHO model, the University of New Mexico, so that our project findings can be easily disseminated across the 240 US institutions offering ECHO. We will also create a dissemination package with curriculum, data collection instruments, and an operations manual to facilitate the use of this project by other ECHO sites. Equally important, this study is disseminable because it was designed using the RE-AIM framework. For instance, understanding aspects of reach, adoption, implementation, and maintenance will assist potential new implementers to assess how amenable the intervention is for their own use. Guided by the RE-AIM framework, we are collecting and evaluating data that are easy to understand and apply in real-world community and clinical settings where research resources are limited. Thus, the RE-AIM framework will greatly strengthen our dissemination capability by providing simplified, pragmatic, user-centered, and theory-driven information to increase the adoption of study findings in additional US nursing home sites.

In conclusion, a multipronged approach to improving infection control and emergency preparedness in nursing homes is critically important, given the toll the COVID-19 pandemic has taken on residents and staff. The ECHO model has significant strengths when compared to traditional training in that it allows for remote learning delivered by a multidisciplinary team of experts, and utilizes case discussions that match the context and capacity of nursing homes. Learners can make real-time changes in practice, as participation equips them to make timely and informed health decisions while leveraging the expertise of specialists during this rapidly evolving pandemic. Understanding how evidence-based infection control guidelines can be implemented effectively in nursing homes is urgently needed, which will have an immediate impact now and for future pandemics.

## Abbreviations

AHRQ: Agency for Healthcare Research and Quality
CDC: Centers for Disease Control and Prevention
CMS: Centers for Medicare and Medicaid Services
CPCQ: Change Process Capacity Questionnaire
ECHO: Extension for Community Health Outcomes
GEE: generalized estimating equation
HHS: US Department of Health and Human Services
IHI: Institute for Healthcare Improvement
NIH: National Institutes of Health
ORIC: Organizational Readiness for Implementing Change scale
PAR: Practice Adaptive Reserve scale
PCORI: Patient-Centered Outcomes Research Institute
QOL: quality of life
RCT: randomized controlled trial
RE-AIM: Reach, Effectiveness, Adoption, Implementation, and Maintenance
